# Application of Physiologically Based Pharmacokinetic Model to Delineate the Impact of Aging and Renal Impairment on Ceftazidime Clearance

**DOI:** 10.3390/antibiotics13090862

**Published:** 2024-09-09

**Authors:** Khaled Abduljalil, Iain Gardner, Masoud Jamei

**Affiliations:** Certara Predictive Technologies Division, Certara UK, Level 2-Acero, 1 Concourse Way, Sheffield S1 2BJ, UK

**Keywords:** ceftazidime, elderly, renal impairment, PBPK model, renal, GFR

## Abstract

The impact of physiological changes during aging on drug disposition has not always been thoroughly assessed in clinical studies. This has left an open question such as how and to what extent patho- and physiological changes in renal function can affect pharmacokinetics in the geriatric population. The objective of this work was to use a physiologically based pharmacokinetic (PBPK) model to quantify the impact of aging and renal impairment (RI) separately and together on ceftazidime pharmacokinetics (PK). The predicted plasma concentrations and PK parameters from the PBPK model were compared to the observed data in individuals of different ages with or without RI (16 independent studies were investigated in this analysis). Apart from clearance in one study, the predicted ceftazidime PK parameters of young adults, elderly, and in individuals with different levels of renal function were within 2-fold of the observed data, and the observed concentrations fell within the 5th–95th prediction interval from the PBPK model simulations. The PBPK model predicted a 1.2-, 1.5-, and 1.8-fold increase in the plasma exposure (AUC) ratio in individuals aged 40, 60, and 70 years old, respectively, with normal renal function for their age compared to 20-year-old individuals with normal renal function. The impact of RI on ceftazidime was predicted to be less marked in older individuals (a 1.04-, 1.43-, and 2.55-fold change in mild, moderate, or severe RI compared to a healthy age-matched control) than in younger individuals (where a 1.47-, 2.03-, and 3.50-fold increase was predicted in mild, moderate, or severe RI compared to a healthy age-matched control). Utilization of the applied population-based PBPK approach allows delineation of the effects of age from renal disease and can better inform future study design and dosing recommendations in clinical study of elderly patients depending on their age and renal function.

## 1. Introduction

The elderly population (aged 65 and over) represents 12.5% of the worldwide population, with this percentage predicted to reach 20% by 2050 [1]. This population is a major consumer of therapeutic drugs, with about 30% of prescribed drugs being consumed by elderly people. Nursing home patients have been reported to be taking from three to more than ten drugs per day [2,3]. Furthermore, the prevalence of use of potentially inappropriate medications among elderly outpatients has been reported to be 36.7% [4]. Due to multimorbidity with different disorders, polypharmacy is common in elderly patients, especially in those living with frailty. Inappropriate use of multiple medications can occur for a variety of reasons, including the lack of evidence-based benefit, drug interaction, adverse drug events, associated risks outweighing the benefit, or failure to adhere to the treatment [4,5]. Early clinical trials often exclude elderly people, posing challenges to determine whether elderly people respond differently from younger individuals [6]. Regulators have released guidance documents on the practical, logistical, and ethical challenges for clinical evaluations of medicinal products in geriatric populations [7,8,9].

Pharmacokinetics (PK) during aging can be affected by a variety of time-varying physiological parameters [10] in addition to the physiological co-variates that affect drug disposition in young healthy adults [11]. Extrapolation of dosing recommendations based on results in young individuals to elderly individuals can carry a level of uncertainty due to absence of information on how these physiological variables can affect drug exposure and response. Knowledge of the physiological changes that occur during aging may help to reduce the uncertainty in extrapolation from young to elderly individuals and may provide insight to potential PK changes and resultant dose adjustments required in elderly individuals.

Many illnesses, such as cancer and cardiovascular, hepatic, and renal diseases are more prevalent in the elderly [12,13]. Aging is associated with progressive structural and functional deterioration of the kidney including age-related reductions in the number of functioning nephrons and glomeruli as well as reduction in renal blood perfusion [14]. These changes lead to continuous reduction in the renal function with age, which in turn is exacerbated in elderly individuals who develop renal impairment (RI) [15,16]. As such, this can increase the exposure and risk of adverse reactions to renally cleared drugs in elderly people [3]. Drug regulators have released detailed guidance on the challenges of evaluating the PK of medicinal products in patents with renal impairment as part of the new drug application process [17,18].

A potentially useful approach to study the interaction between drug properties and physiological factors is the use of physiologically based pharmacokinetic (PBPK) models. This approach provides a means to quantify age-dependent changes in the underlying physiological parameters of the model and changes in these parameters, if accounted for, due to presence of a disease, separately or simultaneously. PBPK models also have the potential to simulate the potential changes in PK (and resultant dose adjustments) that might be observed in an elderly population with comorbidity. Previous work has highlighted the use of PBPK modeling and simulation to guide drug dosing in elderly populations and to explain the increase in exposure of different drugs; however, only a few studies have focused on decreased renal clearance with aging or in individuals of different ages with RI [19,20,21,22,23]. None of these studies aimed to quantify the impact of renal function deterioration due to aging on overall renal function in subjects of different ages.

The objective of this study is to use a previously published PBPK model of ceftazidime [24] to simulate and quantify the impact of changes in renal function due to aging and the presence of RI on ceftazidime PK in elderly and young adult patients without and with different degrees of RI.

## 2. Results

The collated clinical studies assessing the pharmacokinetics of ceftazidime in individuals with different ages and levels of renal function after intravenous administration are described in Table 1. These studies were grouped into studies conducted on “healthy” elderly people [25,26,27,28,29,30,31] and on people with RI [32,33,34,35,36,37,38,39,40]. The virtual trial settings used for replicating clinical studies in healthy elderly and RI people are given in Appendix A.

**Figure 1:**
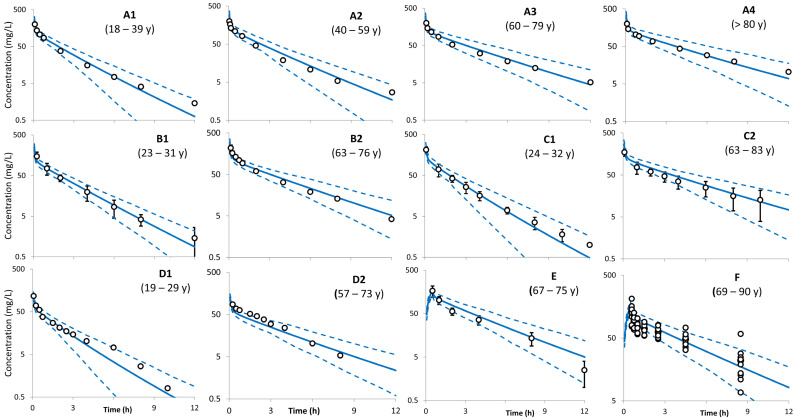
Predicted (vs. observed) ceftazidime PK profiles in young adults and elderly populations after i.v. administration. Solid lines = predicted means; dashed lines = 5th and 95th centiles; circles = observed concentration. All observed data in plots A [26], B [27], C [25], D [30], and E [28] represent the mean profile, except plot F [29] represent individuals. See Section 4/Appendix A for trial settings. Codes A1 to F correspond to those in Appendix A subsection 1 and Table 2.

The PBPK model PK predictions and observed data for ceftazidime in young adult and elderly populations are shown in Figure 1. The simulations were conducted without any further changes to the model or library parameters. The predicted mean concentration profile follows the same shape as the observed mean concentration profiles, and the observed data fell within the simulated 5th–95th prediction interval. Comparison of the predicted PK parameters in elderly people with those available from the clinical studies are shown in Table 2. Additional simulations were executed to predict ceftazidime PK after a bolus of 2 g ceftazidime to four groups of virtual individuals, each of 200 individuals (50% female) aged 25–35, 45–55, 65–75, and 85–95 years.

**Figure 2:**
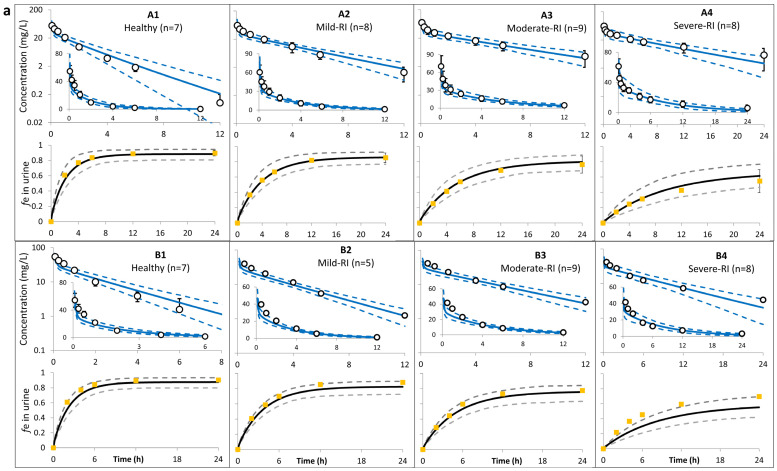

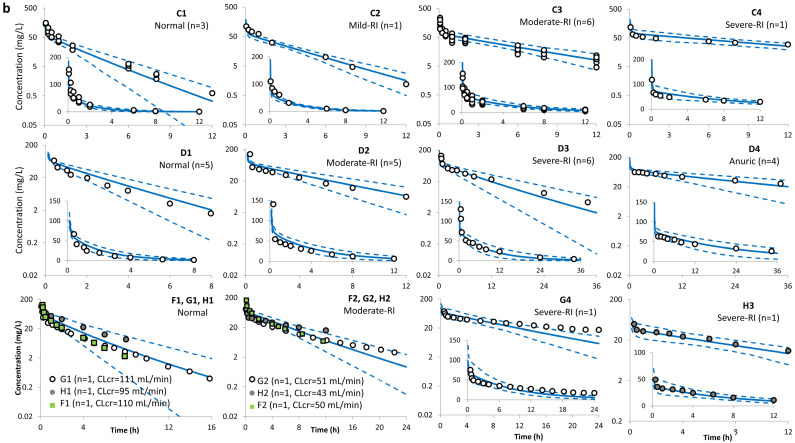
(**a**) Predicted (vs. observed) ceftazidime profiles in adult population with or without renal impairment after i.v. administration of 500 mg. Solid lines = predicted means; dashed lines = 5th and 95th centiles; circles = observed concentration in plasma (plots A [32] and B [33]); squares in plots A [32] and B [33] = observed cumulative fraction of dose recovered in urine as ceftazidime (*f*e). See Section 4/Appendix A for trial settings. Codes A1 to B4 correspond to those in Appendix A sub-section 2 and Table 3. (**b**) Predicted (vs. observed) ceftazidime plasma concentration profiles in adult populations with or without renal impairment after i.v. administration. Solid lines = predicted means; dashed lines = 5th and 95th centiles; circles = observed concentration (plots C [34], D [35], F [40], H [39], and G [38]). See Section 4/Appendix A for trial settings.

Ceftazidime PBPK predictions for adults with normal and impaired renal function are shown in Figure 2a,b together with the observed data from different studies. A comparison of the predicted PK parameters with those available for people with renal impairment from the clinical studies is shown in Table 3.

**Figure 3:**
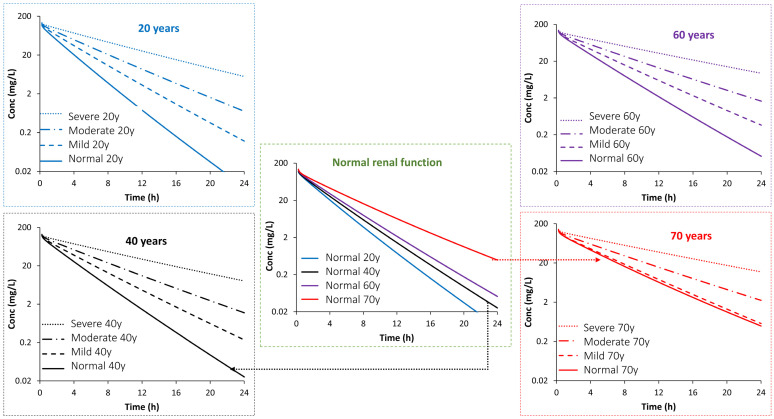
Visual comparison of simulated PK profiles for ceftazidime in healthy individuals (center) with some model outputs at 20 (top left), 40 (bottom left), 60 (top right), and 70 (bottom right) years of age, with different levels of RI severity. For simplicity, only two arrows were linked from the normal group (center) to their locations in the other plots.

Results from simulations executed for model applications are summarized in Table 4. For better visualization, profiles at 20, 40, 60, and 70 years of age are plotted in Figure 3. These results indicate that the AUC is 1.83-, 1.9-, 2.62-, and 4.67-fold higher at age 70 years in individuals with normal renal function, mild-RI, moderate-RI, and severe-RI, respectively, compared with the AUC in healthy individuals aged 20 years. Likewise, the half-life increased, compared to healthy 20-year-old individuals, 1.73-, 1.87, 2.5-, and 4.3-fold at the age of 70 years compared with individuals with healthy, mild, moderate, and severe renal function, respectively.

When the effect of renal impairment is considered at different ages, the AUC increases (compared to a 20-year-old healthy subject) 1.47-, 2.03-, and 3.50-fold in mild, moderate, and severe renal impairment populations at the age of 20 years. The relative change in the AUC with renal impairment (compared to a healthy 70-year-old individual) is 1.04-, 1.43-, and 2.55-fold, in mild, moderate, and severe RI individuals, respectively, if all these people are at the age of 70 years. 

## 3. Discussion

This work describes the use of PBPK modelling to quantify the impact of renal impairment stage separately from the impact of aging on the pharmacokinetics of ceftazidime. The model performance was first verified by comparing the model predictions to observed clinical data in elderly people with and without renal impairment.

Ceftazidime is a suitable compound to assess renal function as its binding to plasma protein is less than 10%, and the degree of protein binding is independent of concentration [41]. As shown in a previous work [24] as well as in this work, the predicted percentage of ceftazidime excreted in urine in young individuals with normal renal function is about 90% (5th–95th percentiles: 80–92%), which agrees with reported data of 80–90% [24]. The mechanism of excretion is mainly passive filtration, and the plasma protein binding is <10%. CLcr is linearly correlated with the drug clearance [41], and the concomitant administration of probenecid did not alter either the ceftazidime serum levels with time or the urinary recovery rate [33]. A high correlation between CL_R_ and CLcr has been reported in a study with patients of varying degrees of renal impairment (r = 0.92 [38], r = 0.97 [39]) suggesting that CLcr can be used to calculate renal function with respect to ceftazidime clearance. Similar results were reported for GFR and ceftazidime clearance in young and elderly healthy individuals [26]. Information in the drug insert about ceftazidime use states that “This drug is known to be substantially excreted by the kidney, and the risk of toxic reactions to this drug may be greater in patients with impaired renal function. Because elderly patients are more likely to have decreased renal function, care should be taken in dose selection, and it may be useful to monitor renal function” [41].

Model prediction for ceftazidime concentrations in elderly healthy individuals was in strong agreement with observations, as shown in Figure 1 and in Table 2 and Table 4. These results show an increase in the AUC, prolongation of half-life, and a decrease in clearance with age. For example, the AUC increases, respectively, about 1.2-, 1.3-, 1.5-, and 1.8-fold in healthy individuals aged 40, 50, 60, and 70 years compared with healthy individuals aged 20 years. This increased exposure reflects the decreased clearance with age (about a 0.6-fold decrease in clearance in healthy elderly people aged 70 years compared with 20-year-old healthy individuals). Different physiological changes occur during aging and can cause profound PK alterations. Major relevant physiological changes that contributed to the observed increase in ceftazidime exposure include the decreasing renal perfusion and filtration. Since plasma fu is about 0.9, it is unlikely that the slight decrease in plasma protein binding level in the elderly will cause any significant changes to ceftazidime PK compared with the young adult population. The model prediction for the volume of distribution at steady state (Vss) was 0.2 L/kg and did not change with age (elderly/young ratio = 1.0); clinical studies indicated a ratio of 1.0 for healthy elderly and young individuals [25,27]. The predicted prolongation of elimination half-life in elderly vs. young healthy adults can therefore be attributed to the change in the renal function and hence the total clearance (Table 1).

PK predictions in the elderly population were in strong agreement with observed values in clinical studies with different dosing levels (Figure 2 and Table 3). All PK parameters were within two times the observed values, and the predicted 5th and 95th percentiles for the systemic exposure in plasma included the observed mean concentration versus time profiles. The predicted rate and extent of the cumulative fraction of the dose excreted as the unchanged drug agreed with the observed values in healthy and RI individuals (Figure 2a and Table 3). The model does not predict any significant changes in ceftazidime Vss = 0.20 L/kg in populations with different renal function due to its high plasma fu ≥0.9. The predicted stability of Vss agrees with clinical observations [35,37,40].

The model predicted a 1.5-, 2.0-, and 3.5-fold increase of the AUC in 20-year-old individuals with mild, moderate, and severe RI, respectively, compared to same-aged individuals with normal renal function. At the age of 70 years, the AUC increased 1.04-, 1.43-, and 2.55-fold in those with mild, moderate, and severe RI, respectively. This is an attenuation by a factor of almost 0.72, which is not a surprise as renal function at 70 years operated at lower level compared with younger individuals (CL 70 years vs. 20 years) in those with severe RI (CL 1.60 vs. 2.13 L/h) and in healthy individuals (CL 4.13 vs. 7.42 L/h), which also agreed well with the 83% increase in the AUC in healthy individuals aged 70 years compared with individuals aged 20 years (Table 4). The significant impact of RI on ceftazidime exposure has led to dosing recommendations in the drug insert based on the subject’s CLcr or serum creatinine and the infection to be treated [41]. However, quantification of the impact of age separately from the impact of disease status has not been investigated before.

This works extends the study published by Zhou et al. [23] where ceftazidime PK was simulated in individuals from five studies with individuals with varying levels of RI. Here, in the current work, the impact of aging has been assessed, and an additional five studies on RI patients were added, enabling us to quantify the separate and combined effects of age and renal disease on the PK of ceftazidime. The adequate description of the observed changes in ceftazidime exposure in populations of elderly individuals with and without RI, indicated that population databases include adequate integration of physiological and pathological changes associated with the deterioration in renal function.

Collated clinical studies with renally impaired patients included individuals of different ethnicity, i.e., two studies with Japanese populations [32,33], one study with Chinese subjects [36], and six studies with European or white or American populations [30,35,37,38,39,40]. Although ethnic differences in renal function/impairment were not considered explicitly in this study, the PBPK model was able to replicate the observed data in people of different ethnicity, age, and renal function, suggesting that ethnicity does not play a major role in ceftazidime pharmacokinetics. Indeed, the observed PK parameters from the two Japanese studies [32,33] were very similar to the rest of observed PK data in Caucasian studies (Table 3). The absence of impact of ethnicity on ceftazidime PK has been shown in earlier work when the model was developed and applied to these populations [24].

While this work shows the application of the PBPK model in predicting ceftazidime PK in elderly subjects with and without renal impairment, there are limitations to the study. First there are limitations in the available data regarding the demographics for the subgroups of patients, which are key covariates in the determination of renal function. This makes it challenging to simulate the studies with matched individuals (in terms of demographics). Another limitation is that published data were not standard with respect to the CLcr (also the CLiv) unit, i.e., mL/min and mL/min/1.73 m^2^, and did not provide the individual body surface area (BSA), with BSA and CLcr being calculated using different methods [27,32,35,37,38,39]. Most studies had limited sample size, with some studies reporting only ceftazidime concentration profiles for a single subject, while a few studies reported PK parameters but not profiles (see Table 3/Figure 2), especially in severe RI populations. It may be noticed from Table 1 that the model underestimates CL (and overestimates the AUC) reported by Norrby et al. [37] for individuals with normal and impaired renal function; however, those observed CL values are higher (and hence the AUC are lower) than those reported by other investigators. The reasons for the difference in the Norrby et al. study compared to other studies is not clear. Whilst the absolute PK parameters in the Norrby study were not well captured by the model, the relative changes in PK parameters in individuals with varying degrees of RI relative to normal subjects were well predicted. Patients studied by van Dalen et al. were critically ill and on mechanical ventilation [38]. In a population PK study, mechanical ventilation has been found to be a significant covariate, causing an increase in ceftazidime peripheral compartment volume by a factor of 2.5 [42], which may explain the observed relatively longer half-life compared with other studies. However, these data are still predicted within the 2-fold error by the model. Finally, a limitation of the current PBPK model is that it does not account for physiological changes in end-stage renal disease, renal failure, or for individuals having hemodialysis. The availability of such additional features in the future would allow an understanding of the effect of these on the PK of compounds mainly passively eliminated by the kidney.

## 4. Materials and Methods

The Simcyp Simulator V23 (Certara UK, Sheffield, UK) was used to perform all simulations. The input parameters for the ceftazidime PBPK model are given in Table 5. The PBPK model for ceftazidime has been published and verified in populations with normal renal functions [23,24]. Briefly, tissue distribution of ceftazidime was described using a full body PBPK model using predicted tissue partition coefficients (Kps) according to Rodgers and Rowland equations [43] without any further scaling. Ceftazidime elimination was described using (passive) renal clearance (~88% of systemic clearance) and a minor contribution of biliary clearance [24].

The PBPK model was used to predict ceftazidime PK with different dosing regimens in young and elderly people with different degrees of renal function using the relevant population file available within the Simcyp Simulator. For predictions in young healthy population, the “Sim-Healthy-Volunteers” population file was used and for predictions in elderly populations, the “Sim-Geriatric” population file was used. For predictions in RI populations [45], the “Sim-Renal Impaired_Mild”, “Sim-Renal Impaired_Moderate”, and “Sim-Renal Impaired_Severe” files were used for ceftazidime predictions in mild, moderate, and severe RI populations, respectively. For all cases, renal function in the developed model was predicted for each subject using the Cockcroft–Gault method normalized to a body surface area of 1.73 m^2^ [46] without any changes to these populations:

$$\mathrm{GFR}\;\left(\mathrm{mL}/\min/1.73m^{2}\right)=\frac{\left(140-\mathrm{age}\right)\;\cdot \mathrm{WT}}{72\;\cdot \mathrm{Scr}/88.42}\;\cdot \frac{1.73}{\mathrm{BSA}}\cdot \left(0.82\;\mathrm{if}\;\mathrm{female}\right)$$

where age, WT, Scr, and BSA, are the individual ages in years, weight in kg, serum creatinine in µmol/L, and body surface area in m^2^.

A total of 20 virtual trials with 10 virtual individuals in each trial (total of 200 virtual individuals matched for age and proportion of females in the actual study) were used to replicate reported settings in published clinical studies. Where the proportion of female in the clinical study was missing, the default proportion for the relevant population within the Simulator was assumed. If the clinical study did not report demographics for subgroups, the given demographics for the overall individuals/patients in that study were used to make PK predictions for each subgroup. Where the stage of RI was not reported in the clinical study, the population file that matched the reported creatinine clearance (CLcr) was used; normal, mild RI, moderate RI, and severe RI were used if the reported CLcr was ≥90, 60–90, 30–60, and 15–30 mL/min/1.73 m^2^ [47] or in units of mL/min [17,18], respectively. No further adjustments were made to population libraries, apart from age and sex to match the clinical studies.

Assessment criteria: The adequacy of the prediction was assessed via comparing the observed data collated from different clinical studies, depending on data availability, against the predicted PK profiles, and/or PK parameters. The PBPK model predictions were considered successful and acceptable if the observed PK profile was fallen within the 95th and 5th percentile of predicted data and the predicted PK parameters were fallen within 0.5 to 2 times the observed data.

Model application: To quantify the impact of different degrees of renal insufficiency at different ages on the ceftazidime exposure, the ceftazidime PBPK model was used to predict exposure after a single bolus dose (2 g ceftazidime) was given to male individuals at age 20, 30, 40, 50, 60, and 70 years with varying levels of RI. Each of these populations consisted of 200 subjects who were either normal, mild-RI, moderate-RI, or severe-RI with respect to their renal function (800 subjects per age group in total).

## 5. Conclusions

A PBPK modelling approach was adopted to quantify the impact of age and renal disease stage separately and in combination using ceftazidime after intravenous administration as a model compound. This goal was achieved by first verifying a PBPK model for ceftazidime in young and elderly without or with RI at different stages of renal insufficiency. The ceftazidime PBPK model presented here adequately predicted the clinical observations of increased and prolonged serum levels of ceftazidime and decreased urinary recovery rate, which mirror the natural reduction of creatinine clearance in healthy elderly people and the additional deterioration of kidney function in the RI patients. The use of PBPK simulation allows prediction of drug exposure and PK parameters in various scenarios, which, in turn, can inform the design of future studies and dose modification in these vulnerable populations.

## Figures and Tables

**Table 1:** Summary of reported demographics in clinical studies investigating ceftazidime PK in the elderly.

Study (Dose)	Age (Years)	Sample Size	Sex	Weight (kg)	CLcr (mL/min)	SerCr (µmol/L)	Additional Notes
(1) Ceftazidime PK studies in elderly individuals							
Ljungberg et al. [26] (2 g i.v. bolus)	18–39	7	5M/2F	NA	112 ± 19	74.3 ± 17.7	GFR determined by ^15^Cr-EDTA; unit of mL/min/1.73 m^2^
	40–59	8	4M/4F	NA	105 ± 26	71.6 ±17.7	
	60–79	13	10M/3F	NA	79 ± 18	90.2 ± 19.5	
	84 ± 3.6	9	5M/4F	NA	56 ± 16	92.0 ± 19.5	
Ljungberg et al. [27] (2 g i.v. bolus)	23–31	9	M	NA	101 ± 6.5	84.9 ± 10.6	GFR determined by ^15^Cr-EDTA; unit of mL/min/1.73 m^2^
	63–76	10	M	NA	77 ± 9.8	90.2 ± 11.5	
Naber et al. [25] (2 g i.v. bolus)	24–32	6	3M/3F	54–81	NA	35.4–79.6	
	63–83	13	11M/2F	55–96		61.9–132.6	
LeBel et al. [30] (2 g i.v. bolus)	19–29	12	6M/6F	44–78.5	76.6–124	70.74–88.42	CLcr calculated using Cockcroft–Gault equation; unit of mL/min. Individual demographics available
	57–73	5	M	50–84	56.9–89.8	70.74–97.26	
Deeter et al. [28] (2 g infused over 30 min)	70.7 ± 3.5	6	3M/3F	75 ± 16	55.9 ± 13.5	88.4 ± 26.5	CLcr calculated using Cockcroft–Gault equation; unit of mL/min/1.73 m^2^
Higbee et al. [29] (2 g infused over 30 min)	69–90	10	M	43.6–81.4	24–80	<221.05	Individual demographic available; CLcr calculated using Cockcroft–Gault equation; unit of mL/min
Shimada et al. [31] (1 g i.v. bolus)	68–82	3	2M/1F	35–55	30–70	61.9–88.4	
(2) Ceftazidime PK studies in renal impairment individuals							
Ohkawa et al. [32] (0.5 g bolus)	20–87	7	29 M/10 F	38–79	105.2–133	NA	CLcr determined from endogenous creatinine clearance corrected for a normalized body surface area (per 1.73 m^2^)
		8			63.1–89.1		
		9			30–56.8		
		8			8.3–29.2		
Saito et al. [33] (0.5 g bolus)	NA	7	M	NA	>90	NA	Determination of CLcr not described
		5			60–90		
		9			30–60		
		8			10–30		
		10			<10		
Ackerman et al. [34] (1 g bolus)	26–92	11	7M/4F	NA	6–113	NA	Individual conc and PK data available, but not for sex and weight. Determination of CLcr not described
Leroy et al. [35] (15 mg/kg bolus)	22–31	5	NA	64–78	110–141	NA	CLcr determined from measurement of endogenous creatinine over time
	26–74	5	NA NA NA NA	41–83	39–72.5	NA	
		6			13.8–27		
		4			2.0–12		
		4			Anuric		
Norrby et al. [37] (1 g; 20-min inf)	57–88	14	8M/6F	NA	47–146	54.8–122	No conc profiles. GFR determined (^51^Cr-EDTA Clearance); individual data for PK, CL_EDTA_, demographics reported
Welage et al. [40] (1 g bolus)	30–91	14	12M/2F	57–95	4.5–122.3	88.4–751.6	Individual data for PK, measured CLcr (urine collection), demographics. Conc profiles from 3 individuals only
Van Dalen et al. [38] (1 g bolus)	34–88	20	14M/9F	NA	0–133.8	NA	Individual PK data and CLcr (urine collection) available, but not demographics. Conc profiles from 3 individuals only
Walstad et al. [39] (1 g, but 0.5 g for severe RI patients)	28–89 (26 of them > 75 years)	9	16M/21F	NA	>50	NA	CLcr estimated using Cockcroft and Gault’s method
		10			50–31		
		10			30–16		
		8			5.0–15		
Lin et al. [36] (2 g b.i.d. bolus)	21–74	6	4M/2F	50–65	51–94	NA	CLcr estimated using Bjornsson’s method using serum creatinine, age, and weight
	58–75	8	5M/3F	42–74	10–35		

CLcr = creatinine clearance; SerCr = serum creatinine; F = female; M = male; NA = not available in the original published paper. To convert SerCr from μmol/L to mg/dL, multiply by 0.0113.

**Table 2:** Predicted vs. observed ceftazidime PK parameters in young adult and elderly populations.

Study Design *			AUC (h·mg/L)			Half-Life (h)			Clearance (L/h)			***f*** e_12h (%) **		
Study	Population Age (N)	(Trial Code)	Obs	Pred	Ratio	Obs	Pred	Ratio	Obs	Pred	Ratio	Obs	Pred	Ratio
Ljungberg et al. **[26]** 2 g i.v. bolus	18–39 y (7)	A1	248 ± 61	278 ± 53	1.12	2.0	1.5 ± 0.3	0.74	8.06	7.48 ± 1.5	0.93	84 ± 7	88 ± 4	1.05
	40–59 y (8)	A2	287 ± 121	323 ± 54	1.13	2.0	1.7 ± 0.4	0.84	6.97	6.38 ± 1.21	0.92	85 ± 7.5	88 ± 5	1.04
	**Ratio (**40–59 y/18–39 y**)**		**1.16**	**1.16**	**1.00**	**0.99**	**1.12**	**1.13**	**0.86**	**0.85**	**0.99**	**1.01**	**1.00**	**0.99**
	60–79 y (13)	A3	392 ± 115	481 ± 118	1.23	2.73	2.64 ± 0.65	0.97	5.1	4.4 ± 1.1	0.86	74 ± 14	83 ± 6	1.12
	**Ratio (**60–79 y/18–39 y**)**		**1.58**	**1.73**	**1.09**	**1.35**	**1.77**	**1.31**	**0.63**	**0.59**	**0.93**	**0.88**	**0.94**	**1.07**
	>80 y (9)	A4	536 ± 142	626 ± 164	1.17	3.54	3.08 ± 0.8	0.87	5.73	3.4 ± 0.85	0.59	67 ± 16	82 ± 7	1.22
	**Ratio (**>80 y/18–39 y**)**		**2.16**	**2.25**	**1.04**	**1.75**	**2.07**	**1.18**	**0.71**	**0.45**	**0.64**	**0.80**	**0.93**	**1.17**
Ljungberg et al. **[27]** 2 g i.v. bolus	23–31 y (9)	B1	277 ± 29	291 ± 49	1.05	1.94	1.67 ± 0.27	0.86	7.22 ± 0.8	7.05 ± 1.2	0.98	87 ± 10	87 ± 4.4	1.00
	63–76 y (10)	B2	418 ± 52	503 ± 119	1.20	2.63	2.84 ± 0.63	1.08	4.78	4.19 ± 0.9	0.88	72 ± 8.6	82 ± 6.3	1.14
	**Ratio (**63–76 y/23–31**)**		**1.51**	**1.73**	**1.15**	**1.36**	**1.70**	**1.25**	**0.66**	**0.59**	**0.90**	**0.82**	**0.94**	**1.15**
Naber et al. **[25]** 2 g i.v. bolus	24–32 y (6)	C1	271	270 ± 55	1.0	1.75 ± 0.14	1.4 ± 0.33	0.80	7.38 ± 0.7	7.71 ± 1.6	1.0	87 ± 8.4	89 ± 4.0	1.0
	65–83 y (13)	C2	422	515 ± 128	1.2	2.9 ± 0.5	2.85 ± 0.69	0.98	4.74 ± 1.0	4.1 ± 0.95	0.86	57 ± 18	82 ± 6.9	1.43
	**Ratio (**65–83 y/24–32 y**)**		**1.56**	**1.91**	**1.2**	**1.66**	**2.04**	**1.23**	**0.64**	**0.53**	**0.83**	**0.66**	**0.92**	**0.14**
Le Bel et al. **[30]** 1 g i.v. bolus	19–29 y (12)	D1	134 ± 13	133 ± 27	0.99	1.9 ± 0.3	1.41 ± 0.35	0.74	7.50 ± 0.7	7.86 ± 1.6	1.05	77 ± 8.6	89 ± 4.3	1.16
	57–73 y (5)	D2	224 ± 79	224 ± 54	1.00	1.9 ± 0.7	2.54 ± 0.58	1.34	4.99 ± 2.0	4.71 ± 1.1	0.94	76 ± 13	86 ± 5	1.14
	**Ratio (**19–29 y/19–29 y**)**		**1.67**	**1.68**	**1.0**	**1**	**1.8**	**1.8**	**0.67**	**0.60**	**0.90**	**0.98**	**0.97**	**0.98**
Deeter et al. **[28]** 2 g infusion	70.7 ± 3.5 y (6)	E	409 ± 62	483 ± 107	1.18	3.7 ± 2.0	2.47 ± 0.59	0.67	4.89 ± 0.80	4.34 ± 0.92	0.89	NA	84 ± 6.2	NA
Higbee et al. **[29]** 2 g infusion	69–91 y (10)	G	463 ± 209	541 ± 137	1.17	3.9 ± 1.3	3.0 ± 0.65	0.77	4.9 ± 1.4	3.93 ± 0.96	0.80	71 ± 3	70 ± 9	0.98
Shimada et al. **[31]** 1 g i.v. (bolus)	68–82 y (3)	F	287 ± 93	260 ± 69	0.91	3.7 ± 1.1	2.76 ± 0.10	0.74	NA	4.08 ± 0.97	NA	71 ± 3	70 ± 9	0.99
Model Predictions 2 g (bolus)	25–35 y (200)	H1		282 ± 55			1.5 ± 0.4			7.4 ± 1.6			88 ± 4	
	45–55 y (200)	H2		328 ± 49			1.8 ± 0.4			6.2 ± 1.1			88 ± 4	
	**Ratio (**45–55/25–35 y**)**			**1.16**			**1.18**			**0.85**			**1.0**	
	65–75 y (200)	H3		499 ± 134			2.5 ± 0.6			4.3 ± 1.1			84 ± 6	
	**Ratio (**65–75 y/25–35 y**)**			**1.77**			**1.69**			**0.58**			**0.95**	
	85–95 y (200)	H4		722 ± 205			3.5 ± 1.0			3.0 ± 0.8			80 ± 8	
	**Ratio (**85–95 y/25–35 y**)**			**2.56**			**2.36**			**0.40**			**0.91**	

* See Table 1 for more details on the demographics and trial settings. N = sample size in the observed study; ** except for Le Bel et al. [30] values, which were for 24 h; NA = not mentioned; AUC is the area under the plasma curve, calculated to infinity; *f*e is the percentage of drug excreted in urine; Trial Code represents the simulated trial design (see Appendix A sub-section 1).

**Table 3:** Predicted vs. observed ceftazidime PK parameters in populations with normal (Healthy-Pop) or impaired (RI-Pop) renal function.

Ref.	Population *; Age (n: CLcr (mL/min)	Trial Code	AUC (h · mg/L)			Half-Life (h)			Clearance (L/h)			*f*e_24h (%)		
			Obs	Pred	Ratio	Obs	Pred	Ratio	Obs	Pred	Ratio	Obs	Pred	Ratio
Ohkawa et al. [32] (0.5 i.v. g bolus)	20–65 y (7 Normal: 105.2–133 ^a^)	A1	72.9 ± 14	71.3 ± 14	0.98	1.55 ± 0.3	1.52 ± 0.4	0.98	8.2 ± 1.5	7.3 ± 1.4	0.89	90 ± 4	89 ± 4	0.99
	20–87 y (8 Mild RI: 63.1–89.1)	A2	133 ± 14	134 ± 15	1.0	2.9 ± 0.6	2.9 ± 0.6	1.0	4.6 ± 1.2	3.8 ± 0.4	0.82	85 ± 6	86 ± 5	1.0
	**Ratio (Mild/Normal)**		**1.83**	**1.88**	**1.0**	**1.84**	**1.93**	**1.1**	**0.56**	**0.52**	**0.92**	**0.95**	**0.97**	**1.0**
	20–87 y (9 Moderate RI: 30–57)	A3	192 ± 32	192 ± 32	1.0	3.9 ± 0.9	4.1 ± 0.9	1.0	2.9 ± 1.0	2.7 ± 0.4	0.92	76 ± 11	80 ± 6	1.04
	**Ratio (Moderate/Normal)**		**2.64**	**2.69**	**1.0**	**2.54**	**2.67**	**1.1**	**0.36**	**0.37**	**1.0**	**0.85**	**0.90**	**1.1**
	20–87 y (8 Severe RI: 8.3–29.2)	A4	338 ± 65	344 ± 66	1.0	6.7 ± 1.8	6.9 ± 1.9	1.0	1.5 ± 0.6	1.5 ± 0.3	1.0	55 ± 15	62 ± 10	1.1
	**Ratio (Severe/Normal)**		**4.64**	**4.82**	**1.0**	**4.34**	**4.51**	**1.0**	**0.18**	**0.21**	**1.1**	**0.61**	**0.70**	**1.1**
Saito [33] (0.5 g i.v. bolus)	20–50 y (7 Normal: >=90)	B1	NA	73 ± 13	NA	1.7	1.7 ± 0.3	1.00	NA	7.0 ± 1.2	NA	90	89 ± 4	0.99
	20–50 y (5 Mild RI: 60–90)	B2	NA	111 ± 11	NA	2.3	2.7 ± 0.4	1.16	NA	4.6 ± 0.5	NA	88	83 ± 5	0.94
	**Ratio (Mild/Normal)**		**NA**	**1.51**	**NA**	**1.4**	**1.57**	**1.16**	**NA**	**0.65**	**NA**	**0.98**	**0.94**	**0.96**
	20–50 y (9 Moderate RI: 30–60)	B3	NA	154 ± 18	NA	3.4	3.6 ± 0.6	1.06	NA	3.3 ± 0.4	NA	78	76 ± 7	0.98
	**Ratio (Moderate/Normal)**		**NA**	**2.10**	**NA**	**2.0**	**2.11**	**1.1**	**NA**	**0.47**	**NA**	**0.86**	**0.87**	**1.0**
	20–50 y (8 Severe RI: 10–30)	B4	NA	267 ± 49	NA	6.1	6.2 ± 1.4	1.02	NA	1.9 ± 0.4	NA	70	55 ± 9	0.79
	**Ratio (Severe/Normal)**		**NA**	**3.64**	**NA**	**3.6**	**3.65**	**1.0**	**NA**	**0.28**	**NA**	**0.77**	**0.63**	**0.82**
Ackerman et al. [34] (1 g i.v. bolus)	26–27 y (3 Normal: >=90)	C1	133 ± 28	138 ± 27	1.0	1.3 ± 0.1	1.5 ± 0.3	1.14	7.8 ± 1.5	7.5 ± 1.4	0.96	NA	88 ± 4	NA
	33–74 y (5 Moderate RI: 34–45)	C3	336 ± 64	353 ± 56	1.16	4.7 ± 2	3.8 ± 0.8	0.87	3.1 ± 0.6	2.9 ± 0.4	0.94	NA	72 ± 7	NA
	**Ratio (Moderate/Normal)**		**2.52**	**2.56**	**1.12**	**3.55**	**2.52**	**0.71**	**0.40**	**0.39**	**0.98**	**NA**	**0.82**	**NA**
Leroy et al. [35] (15 mg/kg i.v. bolus) *	22–31 y (5 Normal: 110–141)	D1	127 ± 15	159 ± 36	1.25	1.6 ± 0.1	1.5 ± 0.3	0.97	7.8 ± 0.8	7.5 ± 1.4	0.96	84 ± 4	88 ± 4	1.05
	26–74 y (5 Moderate RI: 39–73)	D2	314 ± 38	376 ± 90	1.20	3.7 ± 0.8	3.5 ± 0.8	0.92	3.3 ± 0.5	3.2 ± 0.4	0.97	56 ± 7	72 ± 7	1.28
	**Ratio (Moderate/Normal)**		**2.47**	**2.36**	**0.96**	**2.38**	**2.27**	**0.95**	**0.42**	**0.43**	**1.01**	**0.67**	**0.82**	**1.21**
	26–74 y (6 Severe RI: 14–27)	D3	773 ± 119	708 ± 205	0.92	9.3 ± 1.1	6.5 ± 1.7	0.71	1.3 ± 0.1	1.6 ± 0.3	1.25	45 ± 13	63 ± 8	1.42
	**Ratio (Severe/Normal)**		**6.09**	**4.45**	**0.73**	**5.89**	**4.30**	**0.73**	**0.17**	**0.22**	**1.30**	**0.53**	**0.72**	**1.34**
	26–74 y (4 Anuric: 0)	D4	2313 ± 414	2166 ± 849	0.94	25 ± 4.1	19.8 ± 7.3	0.78	0.4 ± 0.0	0.6 ± 0.2	1.42	0.0	0.0	NA
	**Ratio (Anuric/Normal)**		**18.2**	**13.6**	**0.75**	**16.1**	**13.0**	**0.81**	**0.05**	**0.08**	**1.48**	**NA**	**0.00**	**NA**
Norrby et al. [37] (1 g; 20-min i.v. infusion)	57–77 y (6 Normal: 92–146)	E1	118 ± 38	218 ± 54	1.84	1.5 ± 0.4	2.4 ± 0.5	1.57	9.4 ± 3.3	4.9 ± 1.2	0.52	NA	87 ± 5	NA
	69–84 y (5 Mild RI: 60–76)	E2	175 ± 36	264 ± 30	1.51	2.4 ± 0.4	2.7 ± 0.5	1.13	6.0 ± 1.4	3.9 ± 0.5	0.64	NA	87 ± 5	NA
	**Ratio (Mild/Normal)**		**1.48**	**1.21**	**0.82**	**1.60**	**1.15**	**0.72**	**0.64**	**0.79**	**1.24**	**NA**	**1**	**NA**
	62–78 y (3 Moderate RI: 47–54)	E3	228 ± 24	368 ± 64	1.61	3.4 ± 0.3	3.9 ± 0.9	1.14	4.4 ± 0.5	2.8 ± 0.5	0.64	NA	79 ± 7	NA
	**Ratio (Moderate/Normal)**		**1.93**	**1.69**	**0.88**	**2.27**	**1.66**	**0.73**	**0.47**	**0.57**	**1.23**	**NA**	**0.91**	**NA**
Welage et al. [40] (1 g i.v. bolus)	30–36 y (2 Normal: 110–122)	F1	152 ± 37	150 ± 27	0.99	1.7 ± 0.2	1.7 ± 0.3	0.99	7.0 ± 1.7	6.9 ± 1.2	0.98	78 ± 23	87 ± 4	1.12
	49–69 y (5 Moderate RI: 30–60)	F2	336 ± 39	317 ± 45	0.94	3.6 ± 0.5	3.4 ± 0.7	0.93	3.0 ± 0.3	3.2 ± 0.4	1.06	80 ± 15	72 ± 6	0.90
	**Ratio (Moderate/Normal)**		**2.21**	**2.11**	**0.96**	**2.12**	**1.99**	**0.94**	**0.43**	**0.47**	**1.09**	**1.03**	**0.83**	**0.81**
	27–91 y (4 Severe RI: 21–29.5)	F3	582 ± 86	548 ± 89	0.94	6.3 ± 2.4	5.6 ± 1.5	0.89	1.8 ± 0.3	1.9 ± 0.3	1.06	74 ± 11	50 ± 8	0.68
	**Ratio (Severe/Normal)**		**3.83**	**3.65**	**0.95**	**3.71**	**3.31**	**0.89**	**0.25**	**0.27**	**1.09**	**0.95**	**0.57**	**0.61**
Van Dalen et al. [38] (1 g i.v. bolus)	34–88 y (4 Normal: 93–134)	G1	136 ± 36	160 ± 26	1.18	2.5 ± 0.9	1.7 ± 0.4	0.70	7.8 ± 1.7	6.4 ± 1.1	0.83	80 ± 2	89 ± 4	1.11
	34–88 y (3 Mild RI: 72–86)	G2	190 ± 6	268 ± 31	1.41	3.7 ± 1.1	2.9 ± 0.6	0.78	5.3 ± 0.2	3.8 ± 0.5	0.72	88 ± 5	86 ± 5	0.98
	**Ratio (Mild/Normal)**		**1.40**	**1.68**	**1.20**	**1.49**	**1.67**	**1.12**	**0.67**	**0.59**	**0.87**	**1.10**	**0.97**	**0.88**
	34–88 y (4 Moderate RI: 30–59)	G3	393 ± 187	386 ± 66	0.98	6.9 ± 3.1	4.0 ± 0.9	0.58	3.0 ± 1.0	2.7 ± 0.4	0.89	69 ± 10	81 ± 6	1.17
	**Ratio (Moderate/Normal)**		**2.89**	**2.41**	**0.83**	**2.76**	**2.32**	**0.84**	**0.38**	**0.41**	**1.08**	**0.86**	**0.91**	**1.06**
	34–88 y (2 Severe RI: 9–20)	G4	1140 ± 314	681 ± 131	0.60	15.1 ± 1.0	6.9 ± 1.9	0.46	0.9 ± 0.2	1.5 ± 0.3	1.63	41 ± 12	62 ± 9	1.51
	**Ratio (Severe/Normal)**		**8.38**	**4.26**	**0.51**	**6.04**	**3.97**	**0.66**	**0.12**	**0.24**	**1.97**	**0.51**	**0.70**	**1.36**
Walstad et al. [39] (1 g i.v.)	28–89 y (9 Mild RI: ≥50)	H1	232 ± 34	261 ± 32	1.13	2.8 ± 0.5	2.7 ± 0.6	0.95	4.4 ± 0.7	3.9 ± 0.5	0.89	94 ± 8	87 ± 4	0.93
	28–89 y (10 Moderate RI: 31–50)	H2	359 ± 62	382 ± 68	1.06	5.0 ± 1.2	3.8 ± 0.9	0.75	2.9 ± 0.5	2.7 ± 0.5	0.95	80 ± 12	81 ± 6	1.01
	28–89 y (10 Severe RI: 16–30)	H3	279 ± 54	337 ± 65	1.21	8.6 ± 1.7	6.5 ± 1.8	0.75	1.9 ± 0.4	1.5 ± 0.3	0.83	58 ± 5	64 ± 9	1.10
Lin et al. [36] (2 g i.v. b.i.d. bolus)	21–74 y (6 Mild RI: 51–94)	I1	410 ± 13	504 ± 54	1.23	3.3 ± 1.1	2.9 ± 0.6	0.86	5.7	4.0 ± 0.5	0.70	NA		NA
	58–75 y (8 Severe RI: 10–35)	I2	990 ± 265	1114 ± 213	1.13	7.6 ± 1.6	6.2 ± 1.7	0.82	2.0	1.9 ± 0.4	0.97	NA		NA
Model prediction (1 g i.v. bolus)	65–80 y (200 Normal)			254 ± 63			2.9 ± 0.7			4.2 ± 1.0			86 ± 6	
	65–80 y (200 Mild RI)			268 ± 29			3.1 ± 0.5			3.8 ± 0.4			85 ± 5	
	**Ratio (Mild/Normal)**			**1.06**			**1.08**			**0.91**			**0.99**	
	65–80 y (200 Moderate RI)			376 ± 64			4.2 ± 0.8			2.7 ± 0.4			79 ± 6	
	**Ratio (Moderate/Normal)**			**1.48**			**1.46**			**0.66**			**0.92**	
	65–80 y (200 Severe RI)			665 ± 126			7.2 ± 1.7			1.6 ± 0.3			60 ± 8	
	**Ratio (Severe/Normal)**			**2.62**			**2.50**			**0.38**			**0.70**	

* See Table 1 for more details on the demographics and trial settings. NA = not available. AUC was calculated as last AUC_tau_. ^a^ unit is mL/min/1.73 m^2^. Trial Codes represent the simulated trial design given in Appendix A sub-section 2).

**Table 4:** Fold change in PK parameters with age and with increasing renal impairment severity *.

PK Parameter	Age (Years)	Impact of Age or/and Disease Stage (Fold Change from Predicted Mean PK Value in a Population Aged 20 Years with Normal Function)				Impact of Disease Stage (Fold Change from Predicted Mean PK Value in an Age-Matched Population with Normal Function)			
		Normal	Mild RI	Moderate RI	Severe RI	Normal	Mild RI	Moderate RI	Severe RI
Half-Life	20	***1.00***	1.42	1.93	3.22	***1.0***	1.42	1.93	3.22
	30	1.08	1.52	2.07	3.57	***1.0***	1.41	1.92	3.31
	40	1.18	1.59	2.16	3.75	***1.0***	1.36	1.84	3.19
	50	1.30	1.67	2.26	3.96	***1.0***	1.29	1.74	3.06
	60	1.44	1.72	2.32	3.96	***1.0***	1.19	1.61	2.75
	70	1.73	1.87	2.50	4.32	***1.0***	1.08	1.44	2.49
AUC_INF_	20	***1.00***	1.47	2.03	3.50	***1.0***	1.47	2.03	3.50
	30	1.07	1.52	2.12	3.71	***1.0***	1.42	1.98	3.47
	40	1.17	1.58	2.19	3.84	***1.0***	1.34	1.87	3.27
	50	1.31	1.64	2.31	4.04	***1.0***	1.26	1.77	3.10
	60	1.50	1.74	2.41	4.18	***1.0***	1.16	1.60	2.79
	70	1.83	1.90	2.62	4.67	***1.0***	1.04	1.43	2.55
Clearance	20	***1.00***	0.67	0.48	0.29	***1.0***	0.67	0.48	0.29
	30	0.93	0.64	0.46	0.27	***1.0***	0.69	0.50	0.29
	40	0.85	0.62	0.45	0.26	***1.0***	0.73	0.53	0.31
	50	0.76	0.60	0.43	0.25	***1.0***	0.78	0.56	0.33
	60	0.67	0.56	0.41	0.24	***1.0***	0.85	0.62	0.36
	70	0.56	0.52	0.38	0.22	***1.0***	0.93	0.68	0.39

* Reference values are shown in italic bold. PK parameters are relative, hence dimensionless.

**Table 5:** Ceftazidime PBPK model input parameters (see [23,24]).

Parameter	Value	Reference
Physicochemical properties and binding		
Molecular Weight (g/mol)	546.580	Zhou et al., 2019 [23]
Log P	−3.750	
Compound Type	Diprotic Acid	
pKa 1	2.430	
pKa 2	2.890	
BP	0.550	Default
Plasma *f*u (Binding Protein)	0.9 (Human Serum Albumin)	Predicted and used as input
Distribution		
Distribution Model	Full PBPK Model	
Vss (L/kg)	0.20	(Predicted using Method 2 after [43])
Kp Scalar	1.0	
Elimination		
Elimination option	Enzyme Kinetics	
CL_R_ (L/h)	6.0	Zhou et al., 2019 [23]
Biliary CLint (µL/min/million hepatocyte)	0.085 (30% CV)	Adjusted to recover Harding et al., 1983 [44]

LogP = neutral species octanol: buffer partition coefficient; pKa = −log acid dissociation constant; BP = blood-to-plasma partition ratio; *f*u = fraction unbound; CL_R_ = renal clearance; CLint = intrinsic clearance.

## Data Availability

The data presented in this study are available upon written request from the corresponding authors. Workspaces used to generate these results are available on request and can be found in the Certara Members Area.

## Appendix A

1. The following virtual trial settings were used to replicate studies investigating ceftazidime PK in young adult and elderly subjects after i.v. administration.

**Trial (Ref.)**	**Trial Code: Virtual Trial Settings**
Trial A [26]	Trial A1: Healthy-Pop (18–39 years): a bolus of 2 g to 200 individuals (40% female) Trial A2: Healthy-Pop (40–59 years): a bolus of 2 g to 200 individuals (50% female) Trial A3: Healthy-Pop (60–79 years): a bolus of 2 g to 200 individuals (23% female) Trial A4: Healthy-Pop (80–95 years): a bolus of 2 g to 200 individuals (44% female)
Trial B [27]	Trial B1: Healthy-Pop (23–31 years): a bolus of 2 g to 200 individuals (0% female) Trial B2: Healthy-Pop (63–76 years): a bolus of 2 g to 200 individuals (0% female)
Trial C [25]	Trial C1: Healthy-Pop (24–32 years): a bolus of 2 g to 200 individuals (0.5% female) Trial C2: Healthy-Pop (65–83 years): a bolus of 2 g to 200 individuals (0.15% female)
Trial D [30]	Trial D1: Healthy-Pop (19–29 years): a bolus of 1 g to 200 individuals (50% female) Trial D2: Healthy-Pop (57–73 years): a bolus of 1 g to 200 individuals (0% female)
Trial E [28]	Trial E: Healthy-Pop (67–75 years): a 30-min infusion of 2 g to 200 individuals (50% female)
Trial F [29]	Trial F: Healthy-Pop (69–91 years): a 30-min infusion dose of 2 g to 200 individuals (0% female).
Trial G [31]	Trial G: Healthy-Pop (68–82 years): a bolus of 1 g to 200 individuals (0.33% female)

2. The following trial designs were used for predicting ceftazidime PK in individuals with varying degrees of renal impairment after i.v. administration.

**Trial (Ref.)**	**Trial Code: Virtual Trial Settings**
Trial A (Ohkawa [32])	Trial A1: Healthy-Pop: a bolus of 0.5 g to 200 individuals (43% female) aged 20–65 years, with CLcr of 105–133 mL/min/1.73 m^2^. Trial A2: Mild-RI-Pop: a bolus of 0.5 g to 200 individuals (43% female) aged 20–87 years, with CLcr of 63–89 mL/min/1.73 m^2^. Trial A3: Moderate-RI-Pop: a bolus of 0.5 g to 200 individuals (43% female) aged 20–87 years, with CLcr of 30–57 mL/min/1.73 m^2^. Trial A4: Severe-RI-Pop: a bolus of 0.5 g to 200 individuals (43% female) aged 20–87 years, with CLcr of 8–29 mL/min/1.73 m^2^.
Trial B (Saito [33])	Trial B1: Healthy-Pop: a bolus of 1 g to 200 individuals (0% female) aged 20–50 years, with CLcr of >90 mL/min. Trial B2: Mild-RI-Pop: a bolus of 1 g to 200 individuals (0% female) aged 20–50 years, with CLcr of 60–89 mL/min. Trial B3: Moderate-RI-Pop: a bolus of 1 g to 200 individuals (0% female) aged 20–50 years, with CLcr of 30–59 mL/min. Trial B4: Severe-RI-Pop: a bolus of 1 g to 200 individuals (0% female) aged 20–50 years, with CLcr of 10–29 mL/min.
Trial C (Ackerman [34])	Trial C1: Healthy-Pop: a bolus of 1 g to 200 individuals (40% female) aged 26–27 years, with CLcr of 97–113 mL/min. Trial C2: Mild-RI-Pop: a bolus of 1 g to 200 individuals (40% female) aged 27 years with, CLcr of 75 mL/min. Trial C3: Moderate-RI-Pop: a bolus of 1 g to 200 individuals (40% female) aged 33–74 years with, CLcr of 34–45 mL/min. Trial C4: Severe-RI-Pop: a bolus of 1 g to 200 individuals (40% female) aged 78 years with, CLcr of 6 mL/min using lowest limit CLcr of 15 mL/min).
Trial D (Leroy [35])	Trial D1: Healthy-Pop: a bolus of 15 mg/kg to 200 individuals (32% female) aged 22–31 years, with CLcr of 110–141 mL/min. Trial D2: Moderate-RI-Pop: a bolus of 15 mg/kg to 200 individuals (32% female) aged 26–74 years, with CLcr of 39–73 mL/min. Trial D3: Severe-RI-Pop: a bolus of 15 mg/kg to 200 individuals (32% female) aged 26–74 years, with CLcr of 14–27 mL/min. Trial D4: Severe-RI-Pop: a bolus of 15 mg/kg to 200 individuals (32% female) aged 26–74 years, with CL_R_ reset to zero.
Trial E (Norrby [37])	Trial E1: Healthy-Pop: a 20-min infusion of 1 g to 200 individuals (33% female) aged 57–77 years, with CLEDTA 92–146 mL/min/1.73 m^2^. Trial E2: Mild-RI-Pop: a 20-min infusion of 1 g to 200 individuals (60% female) aged 69–84 years, with CLEDTA 60–76 mL/min/1.73 m^2^. Trial E3: Moderate-RI-Pop: a 20-min infusion of 1 g to 200 individuals (33% female) aged 57–77 years, with CLEDTA 47–54 mL/min/1.73 m^2^.
Trial F (Welage [40])	Trial F1: Healthy-Pop: a bolus of 1 g to 200 individuals (0% female) aged 30–36 years, with CLcr of 110–122 mL/min. Trial F2: Moderate-RI-Pop: a bolus of 1 g to 200 individuals (20% female) aged 49–69 years, with CLcr of 34–53 mL/min. Trial F3: Severe-RI-Pop: a bolus of 1 g to 200 individuals (0%female) aged 27–91 years, with CLcr of 21–29.5 mL/min.
Trial G (van Dalen [38])	Trial G1: Healthy-Pop: a bolus of 1 g to 200 individuals (30% female) aged 34–65 years, with CLcr of 93–134 mL/min. Trial G2: Mild-RI-Pop: a bolus of 1 g to 200 individuals (30% female) aged 34–88 years, with CLcr of 72–86 mL/min. Trial G3: Moderate-RI-Pop: an i.v. bolus of 1 g to 200 individuals (30% female) aged 34–88 years, with CLcr of 30–59 mL/min. Trial G4: Severe-RI-Pop: an i.v. bolus of 1 g to 200 individuals (30% female) aged 34–88 years, with CLcr of 9–20 mL/min.
Trial H (Walstad [39])	Trial H1: Mild-RI-Pop: a bolus of 1 g to 200 individuals (57% female) aged 28–89 years, with CLcr ≥50 mL/min. Trial H2: Moderate-RI-Pop: a bolus of 1 g to 200 individuals (57% female) aged 28–89 years, with CLcr 31–50 mL/min. Trial H3: Severe-RI-Pop: a bolus of 0.5 g to 200 individuals (57% female) aged 28–89 years, with CLcr 16–30 mL/min.
Trial I (Lin [36])	Trial I1: Mild-RI-Pop: two 30-min infusions of 2 g each to 200 individuals (33% female) aged 21–74 years, with CLcr of 51–94 mL/min. Trial I2: Severe-RI-Pop: two 30-min infusions of 2 g each to 200 individuals (38% female) aged 58–75 years, with CLcr of 10–35 mL/min.
